# Lesion segmentation on ^18^F-fluciclovine PET/CT images using deep learning

**DOI:** 10.3389/fonc.2023.1274803

**Published:** 2023-12-13

**Authors:** Tonghe Wang, Yang Lei, Eduard Schreibmann, Justin Roper, Tian Liu, David M. Schuster, Ashesh B. Jani, Xiaofeng Yang

**Affiliations:** ^1^ Department of Radiation Oncology and Winship Cancer Institute, Emory University, Atlanta, GA, United States; ^2^ Department of Medical Physics, Memorial Sloan Kettering Cancer Center, New York, NY, United States; ^3^ Department of Radiation Oncology, Icahn School of Medicine at Mount Sinai, New York, NY, United States; ^4^ Department of Radiology and Imaging Science and Winship Cancer Institute, Emory University, Atlanta, GA, United States

**Keywords:** segmentation, PET/CT, neural network, prostate radiotherapy, deep learning

## Abstract

**Background and purpose:**

A novel radiotracer, ^18^F-fluciclovine (anti-3-^18^F-FACBC), has been demonstrated to be associated with significantly improved survival when it is used in PET/CT imaging to guide postprostatectomy salvage radiotherapy for prostate cancer. We aimed to investigate the feasibility of using a deep learning method to automatically detect and segment lesions on ^18^F-fluciclovine PET/CT images.

**Materials and methods:**

We retrospectively identified 84 patients who are enrolled in Arm B of the Emory Molecular Prostate Imaging for Radiotherapy Enhancement (EMPIRE-1) trial. All 84 patients had prostate adenocarcinoma and underwent prostatectomy and ^18^F-fluciclovine PET/CT imaging with lesions identified and delineated by physicians. Three different neural networks with increasing levels of complexity (U-net, Cascaded U-net, and a cascaded detection segmentation network) were trained and tested on the 84 patients with a fivefold cross-validation strategy and a hold-out test, using manual contours as the ground truth. We also investigated using both PET and CT or using PET only as input to the neural network. Dice similarity coefficient (DSC), 95th percentile Hausdorff distance (HD95), center-of-mass distance (CMD), and volume difference (VD) were used to quantify the quality of segmentation results against ground truth contours provided by physicians.

**Results:**

All three deep learning methods were able to detect 144/155 lesions and 153/155 lesions successfully when PET+CT and PET only, respectively, served as input. Quantitative results demonstrated that the neural network with the best performance was able to segment lesions with an average DSC of 0.68 ± 0.15 and HD95 of 4 ± 2 mm. The center of mass of the segmented contours deviated from physician contours by approximately 2 mm on average, and the volume difference was less than 1 cc. The novel network proposed by us achieves the best performance compared to current networks. The addition of CT as input to the neural network contributed to more cases of failure (DSC = 0), and among those cases of DSC > 0, it was shown to produce no statistically significant difference with the use of only PET as input for our proposed method.

**Conclusion:**

Quantitative results demonstrated the feasibility of the deep learning methods in automatically segmenting lesions on ^18^F-fluciclovine PET/CT images. This indicates the great potential of ^18^F-fluciclovine PET/CT combined with deep learning for providing a second check in identifying lesions as well as saving time and effort for physicians in contouring.

## Introduction

Prostate cancer has been estimated to have contributed the most new cancer cases in men in 2021 (1). Prostatectomy is a common approach to treatment of prostate cancer patients. Among patients who have undergone prostatectomy, 20%–40% of them may have disease progression, which is strongly associated with metastases (2). Adjuvant or salvage radiation therapy can then be delivered after prostatectomy depending on risk group, pathology, and prostate-specific antigen (PSA) concentration trend after surgery. The design of a radiation treatment plan requires the identification of disease such that radiation doses can be delivered to include these disease sites. Additionally, accurate definition of these lesions may permit focal dose escalation to further increase tumor control while sparing normal tissue (3).

Current imaging modalities such as CT and bone scans are crucial in the characterization of therapy recurrence. However, they are known to have low diagnostic yield as well as not being useful in lesion delineation for radiation treatment planning (4). MRI, in contrast, provides superior soft tissue contrast for lesion detection and contouring. It has been shown to be effective in detecting local recurrence in the surgical bed but is less useful for metastatic disease (5). Standard ^18^F-FDG PET also has limited utility since prostate cancer can be indolent and not highlighted on the images. Moreover, the excretion of FDG in the bladder may affect the image reading of surrounding anatomy.

Recently, ^18^F-fluciclovine (anti-1-amino-3-[^18^F]fluorocyclobutane-1-carboxylic acid) was approved by the US Food and Drug Administration (FDA) as a novel PET radiotracer with promising diagnostic performance for restaging of biochemically recurrent prostate cancer. As a synthetic amino acid analog with little renal excretion and transport through sodium-dependent and sodium-independent pathways (6–8), ^18^F-fluciclovine has shown higher diagnostic accuracy than conventional imaging studies (9, 10). The improved performance of ^18^F-fluciclovine also enables physicians to make suitable treatment decisions. Studies have reported that, as a result of considering ^18^F-fluciclovine PET/CT images during treatment planning, radiation oncologists changed to salvage radiotherapy management for more than one-third of patients with biochemical recurrence after prostatectomy who first underwent conventional imaging (8). A recent phase 2/3 randomized controlled trial demonstrated that 3-year event-free survival was 63.0% in the conventional imaging group versus 75.5% for the ^18^F-fluciclovine PET/CT group, which means that the inclusion of ^18^F-fluciclovine PET in post-prostatectomy radiotherapy decision-making and planning significantly improved rates of survival free from biochemical recurrence or persistence (11).

One of the basic tasks during the integration of this novel PET radiotracer into radiotherapy planning is lesion identification and delineation, which is key for radiotherapy plan quality and subsequent clinical outcomes, since the therapeutic benefit of radiation therapy relies on high-dose coverage of target volumes while sparing surrounding normal tissues by optimizing beam parameters. Currently, manual contouring on medical images is a routine clinical practice, while in recent years, automatic segmentation methods, especially deep learning-based methods, have become attractive given the time-consuming and observer-dependent nature of manual contouring (12). Although a recent study has reported the feasibility of deep learning in segmenting prostate and dominant intraprostatic lesions on PET images (13), no validated auto-contouring approach has been proposed for ^18^F-fluciclovine PET. Moreover, the task of segmentation of the post-prostatectomy lesion itself is also more challenging, since the possible lesion locations range from the surgical bed to pelvic nodes. In this study, we aimed to investigate the feasibility of our in-house deep learning-based automatic segmentation method for lesion segmentation on the novel ^18^F-fluciclovine PET.

## Materials and methods

### Patients

We retrospectively identified 84 patients enrolled in the Emory Molecular Prostate Imaging for Radiotherapy Enhancement (EMPIRE-1 NCT01666808) trial. The patients from this trial had prostate adenocarcinoma and underwent prostatectomy, with detectable PSA and no systemic metastasis on conventional imaging. Detailed information about the trial can be found in (11). All 84 patients were from Arm B, i.e., receiving radiotherapy directed by conventional imaging plus ^18^F-fluciclovine PET/CT. The median age among the 84 patients was 67 (range 50–83). Institutional review board approval was obtained; informed consent was not required for this Health Insurance Portability and Accountability Act (HIPAA)-compliant retrospective analysis.

The PET/CT images were acquired using a GE Discovery 690 PET-CT scanner (GE Healthcare, Milwaukee, WI, USA) with 10 mCi ^18^F-fluciclovine injected for each patient. PET images were reconstructed using an iterative technique (VUE Point Fx [GE Healthcare]; three iterations, 24 subsets, 6.4-mm filter cutoff) and transferred to a MIM Vista workstation (MIM Software, Beachwood, OH, USA) for interpretation. The reconstructed size of each PET image was 192 × 192 with pixel spacing of 3.646 × 3.646 × 3.270 mm^3^, and that of each CT image was 512 × 512 with spacing of 0.977 × 0.977 × 3.270 mm^3^. Both sets of images were imported into the Velocity AI software (Varian Medical Systems, Palo Alto, CA, USA). Abnormal focal uptake over normal marrow was detected by a nuclear radiologist in the region of prostate beds and extraprostatic sites. A region of interest (ROI) was set around the lesion. A threshold representing the percentage of the maximum standardized uptake value (SUV) in the ROI was applied to the ROI with a 5% increment until the lesion could be clearly identified. The segmented lesion was then reviewed by a physician, who applied necessary fine-tuning; this served as the ground truth contour in this study. For each patient, the PET image volume and lesion contours were then resampled using bilinear interpolation to the size of the CT image volume for the input for network training and testing. Note that these PET-defined contours were not used as final contours in radiation therapy. They were sent to radiation oncologists for use as a reference in defining the final volumes during the treatment planning process. The specific workflow can be seen in our previous publication (14).

### Neural network

In this study, we implemented three different neural networks with increasing levels of complexity (U-net, Cascaded U-net, and a cascaded detection segmentation network) to investigate the general applicability of neural networks in segmenting lesions on ^18^F-fluciclovine PET/CT. U-net is a well-established neural network that has been widely used in a variety of image segmentation tasks (15). It is a fully convolutional neural network that consists of an encoding path and a decoding path with skip connections between them. The encoding path extracts image features in reducing spatial information, and the decoding path upsamples the combined features and spatial information layer by layer.

Cascade U-net is a derivative of U-net. It cascades two U-nets, and the second model uses the features extracted by the first model (16). It applies separate sets of filters for each stage, and therefore its performance is expected to be improved, while GPU memory consumption and training time are increased.

The cascaded detection segmentation network was innovatively developed for this study. It consists of two deep learning-based subnetworks, i.e., localization and segmentation. The localization subnetwork aims to locate the target from the input image and thus reduce the complexity of the segmentation. The segmentation subnetwork then takes the cropped image as input and performs segmentation therein. The PET and/or CT images were first fed into the localization subnetwork to detect the location of the target, i.e., the volumes of interest (VOIs) of lesions. Information on the location of the target, i.e., the center and boundary of the ground truth VOI, was used to supervise the localization subnetwork. The localization subnetwork is implemented *via* a fully convolutional one-stage (FCOS) object detection network. After estimation of the location of the detected VOI, the PET and/or CT images were cropped within the VOIs and then fed into the next segmentation subnetwork. The segmentation subnetwork is a fully end-to-end encoder–decoder network. It is used to generate a binary mask of the input. A combination of Dice and cross-entropy loss is used to supervise the segmentation subnetwork. During inference, with the trained cascaded detection segmentation networks, the model takes the newly arrived image as input and obtains the localization information (VOIs) of the target and the segmentation within the VOIs. Subsequently, based on the localization information, we traced back the segmentation into the original image coordinates to derive the final segmentation.

### Evaluation

The Dice similarity coefficient (DSC) was calculated to quantify the similarity and volume overlap between manual contours and auto-segmentation results. The 95th percentile Hausdorff distance (HD95) was calculated to measure the top 5% of the surface distance between the two contours. Center-of-mass distance (CMD) and volume difference (VD) were used to quantify the difference between the general locations and volumes of the manual contours and segmentation results.

To evaluate the performance of the deep learning methods, we retrospectively investigated 155 lesions from 84 prostate cancer patients who underwent ^18^F-fluciclovine PET/CT scans. Each dataset had lesions contoured by physicians, which served as the ground truth and training targets. The proposed network was trained and evaluated using a fivefold cross-validation strategy among 99 lesions from 58 patients (17) and was further tested on the remaining 26 patients with 56 lesions as a hold-out test. All the test results were analyzed using the aforementioned evaluation metrics and summarized together. The deep learning networks investigated were designed using Python 3.6 and TensorFlow and implemented on an NVIDIA Tesla V100 GPU that had 32 GB of memory. Optimization was performed using the Adam gradient optimizer (18).

In addition to comparing three different networks, for each network, we compared the scenarios of using both PET and CT as input and only using PET as input to investigate the optimal training and testing strategies. We applied the Student’s t-test with a threshold p-value of <0.05 to evaluate the statistical significance of differences between the quantitative metrics in different scenarios.

## Results

**Figure 1:**
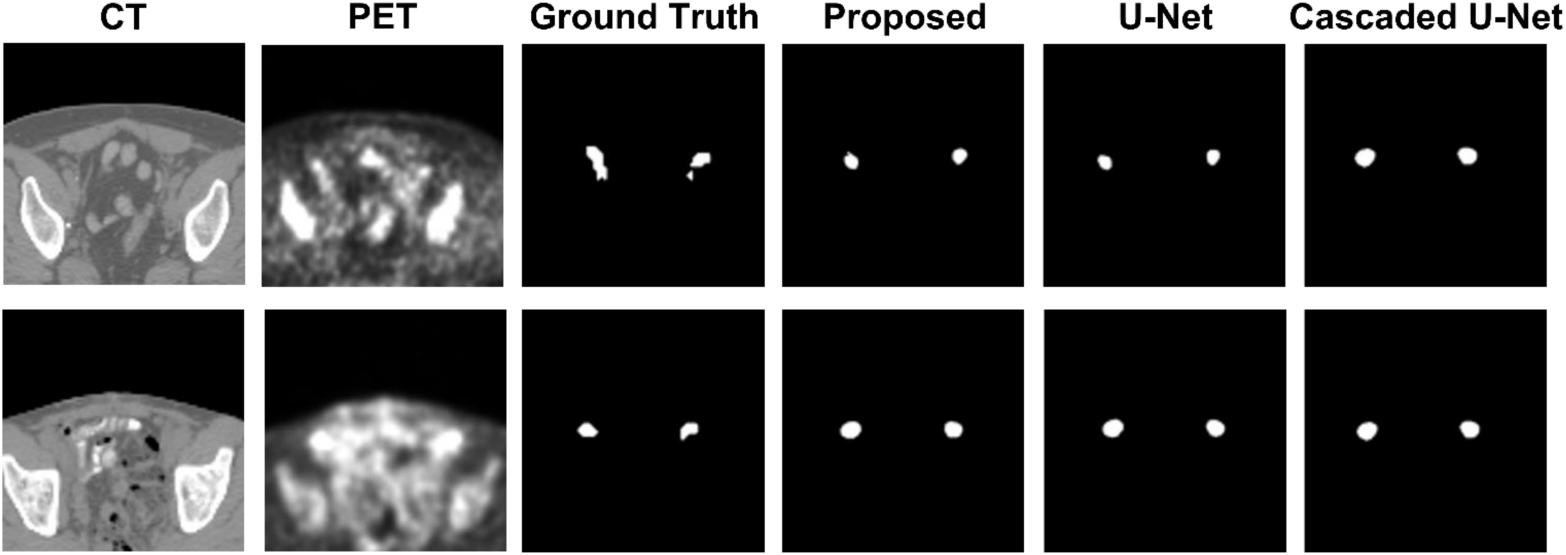
Segmentation results for two illustrative patients. The top row and bottom row show the CT and PET images, along with contours of lesions produced by a physician, the proposed method, U-net, and Cascaded U-net.

**Figure 2:**
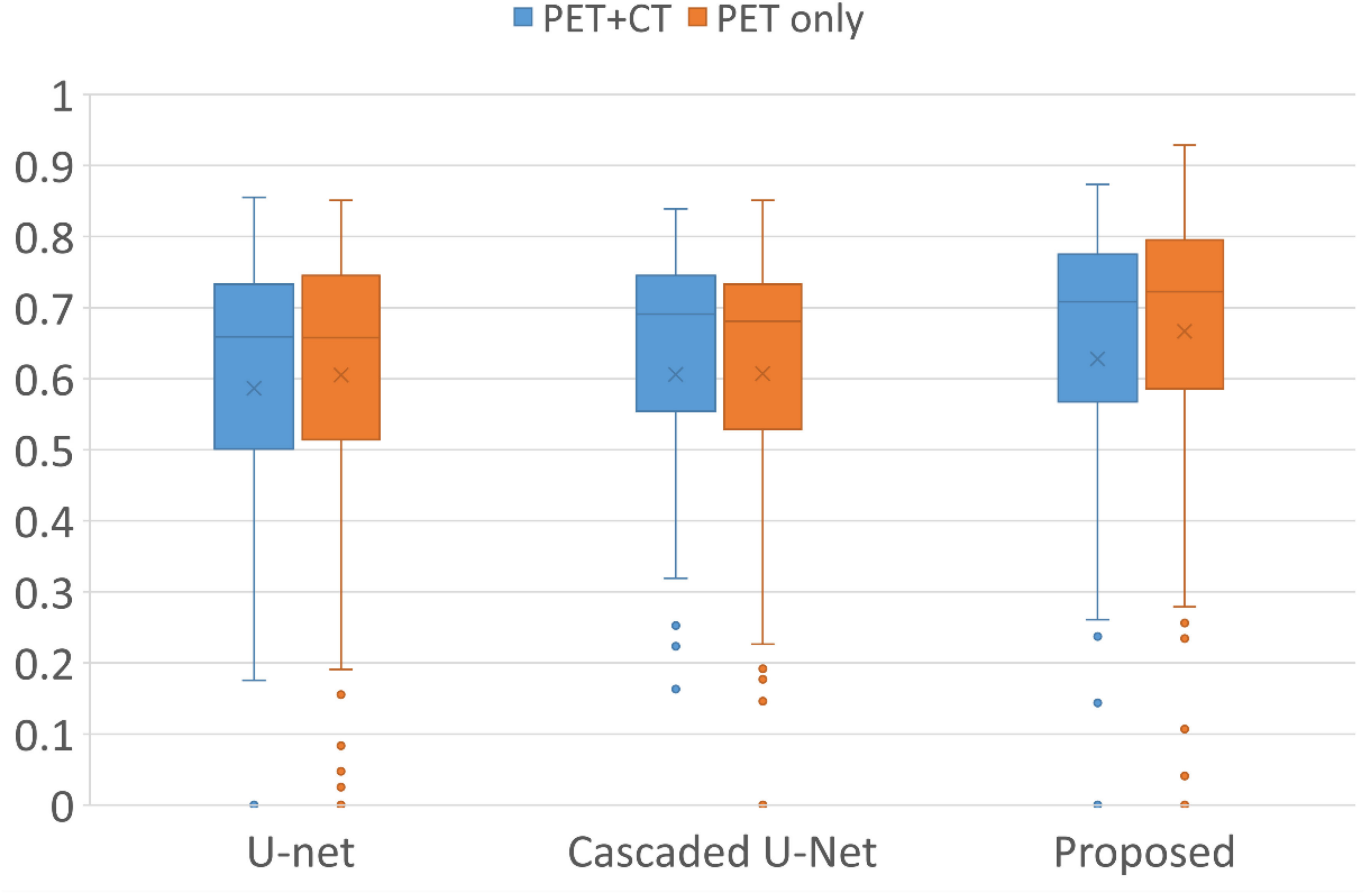
Box plots of the distribution of DSC among all lesions using different networks and inputs. Crosses and dots indicate the mean and outlier data points, respectively. DSC, Dice similarity coefficient.

**Figure 1** demonstrates segmentation results for two illustrative patients. It is seen that all neural networks can generate accurate contours for the lesion when its boundary is clear (below), while a large discrepancy is generated when the boundary is blurred (above). Among the 155 lesions from the 84 patients detected by physicians, all of the three networks successfully detected 153 lesions when only PET served as input for training and testing, compared with 144 lesions detected by all three networks when PET and CT both served as input. **Figure 2** and **Table 1** demonstrate the DSC distribution among the 155 lesions using PET+CT or PET only as input for all three networks. It is shown that the addition of CT led to more failed cases (DSC = 0). It is seen that using PET only resulted in higher median and mean DSC for the proposed method.

**Table 1:** The distribution of DSC among all lesions using different networks and inputs.

	I: U-net		II: Cascade U-net		III: Proposed	
DSC	PET+CT	PET only	PET+CT	PET only	PET+CT	PET only
[0.9, 1]	0	0	0	0	0	1
[0.8, 0.9]	10	15	15	8	24	34
[0.7, 0.8]	46	49	50	57	55	51
[0.6, 0.7]	41	34	43	34	34	27
[0.5, 0.6]	19	20	14	22	11	17
[0.4, 0.5]	16	12	12	8	10	9
[0.3, 0.4]	6	12	6	13	6	9
[0.2, 0.3]	5	6	2	7	3	3
[0.1, 0.2]	1	2	1	4	1	1
(0, 0.1]	0	3	1	0	0	1
0	11	2	11	2	11	2

DSC, Dice similarity coefficient.

After exclusion of all the failed cases, we summarize the statistical results in **Table 2**. In general, neural networks are able to segment lesions with DSC of approximately 0.65 and HD95 of approximately 4 mm on average. The center of mass of the segmented contours deviates from physician-generated contours by approximately 2 mm on average. It is seen that using PET+CT is superior to using PET only for Cascade U-net, while for U-net and the proposed network, any advantage is not statistically significant. Among different networks, we found that the performance of the networks improves with complexity in general. When using PET+CT as input, the proposed method outperforms the other two networks on all the metrics, especially on DSC and VD, with significant differences (p < 0.05).

**Table 2:** Summary of quantitative evaluation metrics for different networks and inputs.

		DSC		HD95 (mm)		CMD (mm)		VD (cc)	
Network Input:		PET+CT	PET only	PET+CT	PET only	PET+CT	PET only	PET+CT	PET only
I: U-net	Mean	0.631	0.613	4.329	4.579	2.261	2.461	0.831	0.802
	Std	0.145	0.183	1.962	2.390	1.520	1.802	0.828	1.158
p-value	PET+CT *vs.* PET only	0.434		0.140		0.389		0.720	
II: Cascade U-net	Mean	0.652	0.615	4.738	4.501	2.160	2.407	0.829	1.003
	Std	0.148	0.169	7.498	2.139	1.522	1.808	1.158	1.188
p-value	PET+CT *vs.* PET only	0.001		0.027		0.193		0.004	
III: Proposed	Mean	0.676	0.675	3.982	4.162	2.003	2.158	0.597	0.679
	Std	0.149	0.171	2.233	2.335	1.466	1.638	0.862	1.069
p-value	PET+CT *vs.* PET only	0.348		0.520		0.614		0.128	
p-value*	I *vs.* II	0.001		0.496		0.327		0.976	
	I *vs.* III	<0.001		0.003		0.005		<0.001	
	II *vs.* III	0.002		0.219		0.082		<0.001	

DSC, Dice similarity coefficient; HD95, 95th percentile Hausdorff distance; CMD, center-of-mass distance; VD, volume difference.

*P-value is calculated for PET+CT only.

**Figure 3:**
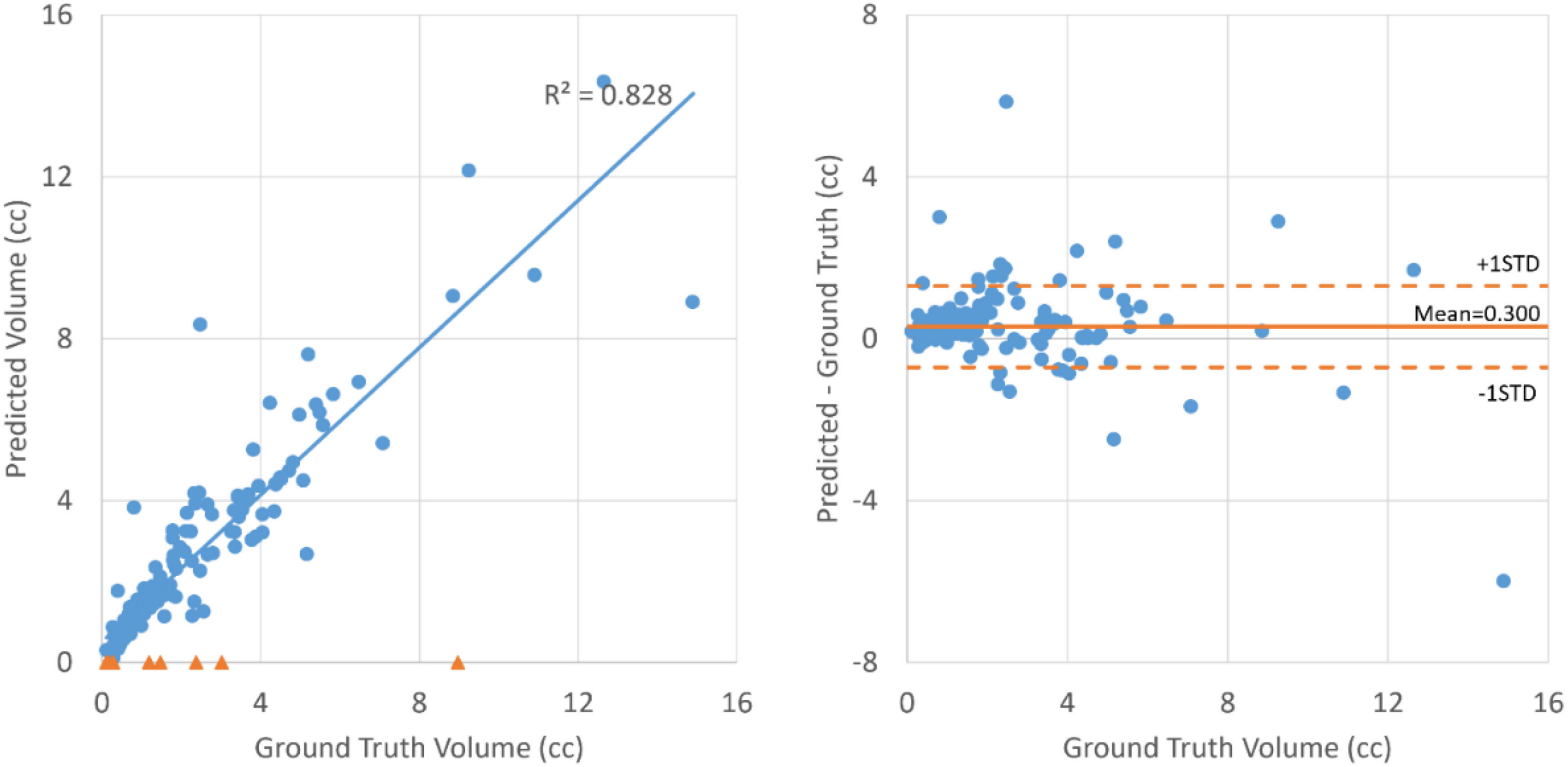
Linear regression (left) and Bland–Altman plot (right) for the volumes of ground truth contours and contours segmented by the proposed method with PET+CT as input. The blue dots represent lesions that are successfully detected. The orange triangles on the left represent lesions that failed to be detected.

As shown in **Table 2**, all the neural networks segmented lesions with volume errors of less than 1 cc on average; the proposed method with PET+CT as input achieved volume errors of less than 0.6 cc. Linear regression and Bland–Altman plots representing the volumes of contours predicted by the proposed method with PET+CT as input are shown in **Figure 3**. The average volumes of segmented contours and ground truth contours were 2.59 cc and 2.29 cc, respectively. A statistically significant (p < 0.05) over-estimation of volume was found; however, the magnitude of over-estimation was minimal (0.3 cc) compared to 2.29 cc, which was the average volume of lesions in this study.

## Discussion

In this study, we investigated the feasibility of using deep learning-based automatic segmentation methods on PET/CT images produced using a novel ^18^F-fluciclovine radiotracer. We trained and tested three different neural networks on 155 lesions of 84 patients with corresponding manual contours. The deep learning methods were shown to detect 144/155 lesions and 153/155 lesions successfully when PET+CT and PET only, respectively, served as input. Quantitative results demonstrated that neural networks were able to segment lesions, with an average DSC of 0.68 ± 0.15 for a novel network proposed by us.

In recent years, there has been active research into the use of ^18^F-fluciclovine as a radiotracer for PET/CT imaging. A recent phase 2/3 clinical trial showed that ^18^F-fluciclovine PET/CT can be used to guide postprostatectomy salvage radiotherapy for prostate cancer in radiotherapy decision-making and planning, with significantly improved rates of survival free from biochemical recurrence or persistence compared to conventional imaging (bone scan and either CT or MRI) (11). However, most lesions are less than 3 cc and are only visible on a small number of slices; thus, they may be neglected. In this study, deep learning methods were able to identify over 90% of lesions. This approach can serve as a redundant check for lesion identification in order to avoid missing targets for treatment. In addition to identification, this method can also delineate lesions to save time for physicians. Moreover, the neural network generates contours more consistently than humans, which can mitigate intra-observer variation. The combination of ^18^F-fluciclovine PET/CT and deep learning methods has great potential in benefiting post-prostatectomy prostate cancer patients.

Compared to other segmentation studies, this study was more challenging due to the small size of the target. The challenges were twofold. For the neural network, a small target means fewer image features presented on the image, which poses difficulty in feature extraction during training and testing. Furthermore, current evaluation metrics may not properly quantify the segmentation results for targets of very small size. A subtle difference, which may not be clinically important, can lead to a large decline in DSC because the difference between two contours can be comparable in magnitude with the target size. The difficulty in segmenting small objects, such as chiasms, has also been reported in other studies (19–22). In this regard, distance-based metrics such as HD95 may be more suitable for small objects, since they directly measure the contour surface distance, which can be critical for small organs. In this study, the HD95 was approximately 4 mm. Note that the pixel spacing of PET images is approximately 3 mm. Thus, the contour surface error was of the magnitude of a pixel.

The addition of CT as input to the neural network contributed to more cases of failure (DSC = 0), and among those cases of DSC > 0, it was shown to have no statistically significant difference with the use of PET only as input for our proposed method. A potential reason is that lesions are not well presented on CT images. The addition of CT images that contain few features of lesions may confuse the network in some cases. However, although a CT image does not provide lesion features, it does provide more anatomy features than PET and may help the network to identify the general location of lesions. However, our method already has a dedicated step for localizing lesions from the entire image volume, which may explain the limited improvement produced by the inclusion of CT. For U-net and Cascade U-net, which do not include the localization step, CT provides additional anatomic information to help locate lesions, and thus, its benefit is clearer.

There are a few limitations to this feasibility study that need to be overcome before the clinical use of deep learning segmentation methods. First, the training and testing datasets in this study were biased toward small-volume lesions. Among the 155 lesions investigated in this study, 115 lesions had a volume of less than 3 cc. Thus, the trained model may be more suitable for small-volume lesions. In order to minimize such bias, it would be desirable to collect data from more patients with large-volume lesions for a more balanced training and testing dataset. In addition, all of the datasets in this study were from the same PET/CT scanner with an identical image reconstruction algorithm. It is unclear how different reconstruction algorithms would affect the performance of the neural networks evaluated in this study. Incorporating datasets from multiple centers with different scanners and reconstruction algorithms would enable further evaluation of the generalizability of the models. Second, although we reported the differences between auto-segmentation results and manual contours in terms of quantitative metrics, their potential clinical impact on plan optimization and treatment outcomes needs further investigation. Moreover, the current ground truth contours are based on the experience of our physicians in selecting the percentage of SUV. More studies are needed to investigate the selection criteria of SUV percentage for this novel radiotracer so that the neural network can be trained with contours closer to the ground truth in order to reduce inter-observer variation. On the other hand, a thorough study of inter-observer variation would also help in understanding the performance of the neural networks. For example, all the neural networks in this study segmented lesions with volume errors of less than 1 cc on average, which seems non-negligible when compared with the average volume of lesions in this study of 2.29 cc. However, it is more desirable to compare this value with inter-observer variation, so that the uncertainty of neural network performance can be compared with human uncertainty. The partial volume effects in PET images may further add systematic errors by reducing the measured maximum SUV (23), for which the contours were drawn on. We are currently also collecting patients with prostate-specific membrane antigen (PSMA) PET imaging scans to train and validate deep learning-based segmentation models. This new FDA-approved scan provides more precise detection of prostate cancer and its migration; thus, its integration with deep learning is worth further investigation.

## Conclusions

We investigated the feasibility of using deep learning-based automatic segmentation methods on PET/CT images produced using a novel ^18^F-fluciclovine radiotracer with 155 lesions from 84 patients. The study demonstrated that deep learning has great potential in segmenting lesions on ^18^F-fluciclovine PET/CT images, with a high detection rate and high delineation quality. Our originally developed neural network was shown to have better performance than current ones used in this task. The use of a deep learning-based auto-segmentation method on ^18^F-fluciclovine PET/CT images would provide a second check in identifying lesions as well as saving time and effort for physicians in contouring.

## Data availability statement

The original contributions presented in the study are included in the article/supplementary material; further inquiries can be directed to the corresponding author.

## Ethics statement

The studies involving humans were approved by the IRB office of Emory University. The studies were conducted in accordance with the local legislation and institutional requirements. The ethics committee/institutional review board waived the requirement for written informed consent for participation from the participants or the participants’ legal guardians/next of kin because this was a retrospective study.

## Author contributions

TW: Data curation, Formal analysis, Methodology, Writing – original draft, Writing – review & editing. YL: Methodology, Writing – review & editing. ES: Resources, Writing – review & editing. JR: Resources, Writing – review & editing. TL: Resources, Writing – review & editing. DS: Conceptualization, Data curation, Resources, Writing – review & editing. AJ: Conceptualization, Data curation, Resources, Writing – review & editing. XY: Conceptualization, Data curation, Funding acquisition, Investigation, Methodology, Resources, Supervision, Writing – review & editing.
